# Coadministration antagonist dopamine receptor D4 with CB2 receptor agonist decreases binge-like intake of palatable food in mice

**DOI:** 10.3389/fnbeh.2025.1572374

**Published:** 2025-04-29

**Authors:** Luis Miguel Rodríguez-Serrano, Ana Paola López-Castillo, María Cristina Cabrera-Mejía, Ana Sofía Cedillo-Figueroa, Nyahn Zepeda-Ortigosa, Carolina Carregha-Lozano, María Elena Chávez-Hernández

**Affiliations:** Facultad de Psicología, Universidad Anáhuac México, Huixquilucan, Estado de México, Mexico

**Keywords:** endocannabinoid system, CB2 receptor, dopamine system, dopamine receptor D4, binge-like intake, palatable food

## Abstract

**Introduction:**

Food intake is regulated by two systems: homeostatic and hedonic. An imbalance between these systems can induce overconsumption, such as binge eating disorder (BED), and is associated with dysregulation of the dopamine reward system. The cannabinoid type 2 receptor (CB2R) has been identified in dopamine neurons and may play an important role in motivated behaviors, including food intake. Nevertheless, the interaction between the dopamine D4 (DRD4) receptor and CB2R in binge-like intake has not yet been identified. Therefore, the present study aims to evaluate the effects of intraperitoneal administration of DRD4 antagonist (L-745870), as well as the coadministration of DRD4 antagonist with either CB2R agonist (HU308) or antagonist (AM630), on binge-like intake of palatable food (PF) in adult male mice.

**Methods:**

We used adult male 34 C57BL6/J mice. All animals were housed individually and had *ad libitum* access to standard diet (SD) and water. To evaluate binge-like intake, the animals had 1 h of access to PF during 12 baseline binge eating test (BET) sessions. Mice were then randomly assigned to the following treatment groups: 1) vehicle; 2) L-745870; 3) L-745870-HU308, 4) L-745870+AM630 to be evaluated under the effect of treatments for three additionally BET sessions.

**Results:**

Our results show that DRD4 antagonist reduced binge-like intake of PF, and that a coadministration with a CB2R agonist induced an even more pronounced reduction of binge-like intake.

**Conclusion:**

These findings suggest an interaction between the dopaminergic and endocannabinoid systems in the modulation of binge-like intake of PF in adult mice, where CB2R activation participates in modulating reward pathways and reducing binge-like behavior.

## 1 Introduction

Palatable food (PF) disrupts appetite regulation by inducing pleasure and reward and contributing to hyperphagia (de Macedo et al., 2016). Furthermore, PF consumption increases the risk of developing binge eating disorder (BED) (Sinclair et al., 2015; Chávez-Hernández et al., 2024), which is characterized by uncontrollable episodes of eating in a short period of time, with a subjective loss of control of overconsumption behavior (De Sa Nogueira et al., 2021; Bourdy and Befort, 2023). In this regard, endocannabinoid system (ECS) impaired activity has been suggested in the pathophysiology of BED (Satta et al., 2018). In this regard, it has been shown that the ECS participates in regulating homeostatic and hedonic food intake pathways (Lau et al., 2017). The ECS comprises the endogenous ligands anandamide (AEA) and 2-arachidonoylglycerol (2-AG), whose effects are mediated by two receptors: Cannabinoid Receptor 1 (CB1R) and Cannabinoid Receptor 2 (CB2R) (Bhattacharjee and Iyer, 2023). AEA and 2-AG are synthesized by postsynaptic neurons in response to neurotransmitter release functioning as negative feedback regulators and inhibiting further neurotransmitter release at excitatory or inhibitory synapses (Coccurello and Maccarrone, 2018; Kurtov et al., 2024). For instance, CB2R activity results in the activation of the Gi/o protein, which is associated with different cellular pathways, including inhibition of adenylate cyclase (AC) (Atwood and Mackie, 2010; Jordan and Xi, 2019) and activation of intracellular kinases, such as PI3K-Akt pathway, and extracellular signal-regulated (ERK) kinases (Ferrisi et al., 2021; Bhattacharjee and Iyer, 2023), which ultimately results in the suppression of neuronal activity.

The dopaminergic system has a key role in the reward value of food (Lewis et al., 2021), and it has been shown to contribute to the development and maintenance of binge intake by driving this maladaptive eating behavior (Yu et al., 2022). Research indicates that PF consumption activates the brain reward circuit, involving mesolimbic dopaminergic pathways from the ventral tegmental area (VTA) to the nucleus accumbens (NAc) and prefrontal cortex (Sharma et al., 2013; Schoukroun et al., 2024). Furthermore, it has been shown that PF consumption induces deficits in the dopamine (DA) reward system (de Macedo et al., 2016), which is associated with increased susceptibility to binge-like eating (Sun et al., 2022). Additionally, it has been demonstrated that PF may induce changes in neuronal plastic changes in the brain’s reward circuitry that lead to overconsumption (Morin et al., 2017). Moreover, research indicates that PF intake increases DA release in NAc and VTA, a key region in the brain’s reward circuit (Arcego et al., 2020; Bourdy et al., 2021; Wallace and Fordahl, 2022). In particular, the dopamine receptor D4 (DRD4) has been identified to be involved in the modulation of the reward process of PF consumption and may contribute to the dysregulation of food intake in patients with eating disorders (Botticelli et al., 2020). In this regard, it has been shown that the administration of DRD4 agonist induces hyperphagia in male rats (Tejas-Juárez et al., 2014), while blocking DRD4 activity decreases the sucrose consumption in rats (López-Alonso et al., 2023). However, the role of DRD4 in binge-like intake of PF remains unknown.

Studies suggest that the ECS can modulate DA activity. For example, the systematic administration of CB1R antagonist decreases DA release in NAc induced by PF intake (Melis et al., 2007), suggesting that the hedonic response to PF depends on the ECS activity, probably through modulation of the mesocorticolimbic system (Satta et al., 2018). In this regard, it has been shown that ECS also participates in BED (Scherma et al., 2013; Bourdy and Befort, 2023), and it has been shown that high-sugar intake is induced by an increased expression of CB2R in the NAc and VTA in adult rats (Bourdy et al., 2021). Furthermore, the administration of CB2R agonist hyperpolarizes dopaminergic neurons of VTA (Zhang et al., 2014). This suggests a potential role of CB2R activity in the modulation of DA release, contributing to sugar consumption. Additionally, the administration of CB2R agonists has been shown to reduce binge intake of PF in adolescent mice (Rodríguez-Serrano and Chávez-Hernández, 2025) and reduce sucrose self-administration in mice adults (Bi et al., 2020). Nevertheless, it is important to evaluate how the interaction of CB2R with DRD4 modulates the consumption of PF in binge-like behavior to gain a deeper understanding of the neurobiology that underlies the regulation of PF consumption and binge-like intake. Therefore, the aim of the present study is to determine the interaction between DRD4 antagonism and CB2R agonism and antagonism in binge-like intake of PF in adult mice.

## 2 Materials and methods

### 2.1 Subjects

Thirty-four adult male C57BL6/J mice, weighing 17.8 g at the start of the experiment (8 weeks old), were used. All animals were individually housed to have a precise measure of food intake per animal. Mice were maintained in a temperature (21 ± 1°C) and humidity-controlled environment under a standard 12 h light–dark cycle, with lights on (ZT0) at 7:00 a.m. and off (ZT12) at 7:00 p.m. All animals had ad libitum access to water and a standard diet (SD; Nutricubos Purina^®^; 3,36 kcal/g; 23.0% protein, 3.0% fat, and 6.0% fiber). Mice also had 1 h access to a PF (chocolate sandwich cookies [Oreo^®^ Nabisco^®^] 4.67 kcal/g, 4.1% protein, 19.2% fat, 69.5% carbohydrates) with intermittent diet protocol to induce binge-like intake (Corwin et al., 2011; Chávez-Hernández et al., 2024). Since the beginning of the experiment, the weight of the mice was measured weekly, and SD consumption was manually recorded every 24 h until the end of the experiment.

### 2.2 Evaluated behavior: binge eating test

Binge eating test (BET) is defined as the overconsumption of PF in a short time under conditions that are not necessarily driven by caloric need (Rodríguez-Serrano and Chávez-Hernández, 2025). Therefore, we evaluated the binge-like intake of PF, and the animals had 1 h access to PF in the home cage. The BET was evaluated with an intermittent model, with the following access to PF days: Monday, Wednesday, and Friday for 1 h (11–12 h) (Chávez-Hernández et al., 2024; Rodríguez-Serrano and Chávez-Hernández, 2025). A total of 15 BETs were conducted in 5 experimental weeks. At the end of each week, the animals were weighted to identify changes in body weight induced by BET. The PF was weighted before and after the 1 h access to register consumption, and caloric intake was calculated as follows for both PF and SD: Caloric intake = (WFfound – WFplaced) x Kcal, where WFfound represents the weight in grams of the food found on the cage, WFplaced is the weight of the food when first placed on the cage, and Kcal is the kilocalories per gram of PF or SD. Additionally, binge-like intake was determined as consuming ≥20% of total daily kilocalories from PF (Muñoz-Escobar et al., 2019); first, total caloric intake (TOTALkcal) was calculated as the sum of kilocalories from PF and calories from SD (TOTALkcal = PFkcal + SDkcal). Afterward, the proportion of PF intake kilocalories (Kcal %) was calculated as follows: PF intake kcal % = (PFkcal/TOTALkcal) × 100.

### 2.3 Body weight analysis

Mean weekly body weight was analyzed to evaluate differences between groups across BET sessions from baseline (BL) week to Week 5. Moreover, differences between groups on body weight were analyzed in Weeks 4 and 5.

### 2.4 Open field test

Locomotor activity and exploratory behaviors were evaluated with the open-field test (OFT). Mice were placed in the center of a 50 cm × 50 cm × 50 cm squared arena and were permitted to explore the open field for 5 min. The behavior of each animal was recorded using a video camera, and locomotor behavior was evaluated by distance traveled in center and periphery zone parameters (Kraeuter et al., 2019a). It is important to note that this task was also used as habituation for the food preference test and Novel Object Recognition Test.

### 2.5 Food preference test

The food preference test (FPT) was used to evaluate preference between SD or PF within an animal exploratory behavior and food interaction paradigm. Mice were placed in an open field with two types of food (standard chow and palatable diet) presented simultaneously for 5 min. The proximity of the mice’s heads to each food type was measured directly. Time exploring each food was analyzed as a parameter for this task and presented as a preference index obtained by dividing time exploring each food by total task time (5 min).

### 2.6 Novel object recognition test

The Novel Object Recognition Test (NORT) was used to evaluate memory in a novelty recognition paradigm. This task consists of three phases: Acquisition, Short-term Memory (STM), and Long-Term Memory (LTM). First, in the acquisition phase, the animal is allowed to explore freely for 5 min in a 50 cm × 50 cm × 25 cm squared arena with two identical objects, equidistant from each other and from the arena’s wall. The STM phase takes place 2 h after the acquisition phase; in STM, one of the objects is replaced by another object completely different in shape, color, and texture, and animals explore freely for 5 min. Finally, 24 h after the acquisition stage, LTM is evaluated by replacing the object used in the STP phase with another object completely different in shape, color, and texture and letting animals explore freely for 5 min. Time exploring each object was analyzed as a parameter for the two stages (STM and LTM) of this task and presented as a preference index obtained by dividing time exploring each object by total task time (5 min).

### 2.7 Forced swim test

The forced swim test (FST) was used to evaluate depression-like behavior and record immobility (Kraeuter et al., 2019b). A glass cylinder (20 cm in diameter and 50 cm in height) was used and filled with water up to 30 cm from the base at a temperature of 24 ± 1°C. The subjects will be placed in the cylinder for 6 min. Time immobile was analyzed as a parameter for this task.

### 2.8 Experimental design and procedure

Animals were distributed in the following treatment groups:

1. CON group (*n* = 6) without access to BET, for which only standard diet intake and weight were measured.
2. VEH group (*n* = 7) with access to BET and administered a vehicle consisting of dimethyl sulfoxide (DMSO) and saline solution (1:9; 1 mL/kg) immediately before BET sessions.
3. L-745870 group (*n* = 7) with access to BET and administration of D4R selective antagonist [5 mg/kg Tocris Bioscience, UK (St Onge and Floresco, 2009), 30 min before BET sessions].
4. L-745870 + HU308 group (*n* = 7) received the same treatment as the L-745870 group and coadministration of CB2R selective agonist immediately before BET sessions (5 mg/kg; Sigma-Aldrich, St. Lous MO) (Schmitz et al., 2016).
5. L-745870 + AM630 group (*n* = 7) received coadministration of L-745870 (5 mg/kg) and CB2R selective antagonist (5 mg/kg; Sigma-Aldrich, St. Lous MO) (Verty et al., 2015), both treatments 30 min before BET sessions.

All drugs were prepared by dilution in a 9:1 vehicle consisting of one unit of DMSO and nine units of sterile saline solution.

Animals were presented with 12 baseline sessions of BET and subsequently were randomly assigned to one of the five groups (CON, VEH, L-745870, L-745870 + HU308, or L-745870 + AM630) and were evaluated for three additional BET sessions (13, 14, and 15), where they received intraperitoneal administration of treatments.

**Figure 1:**
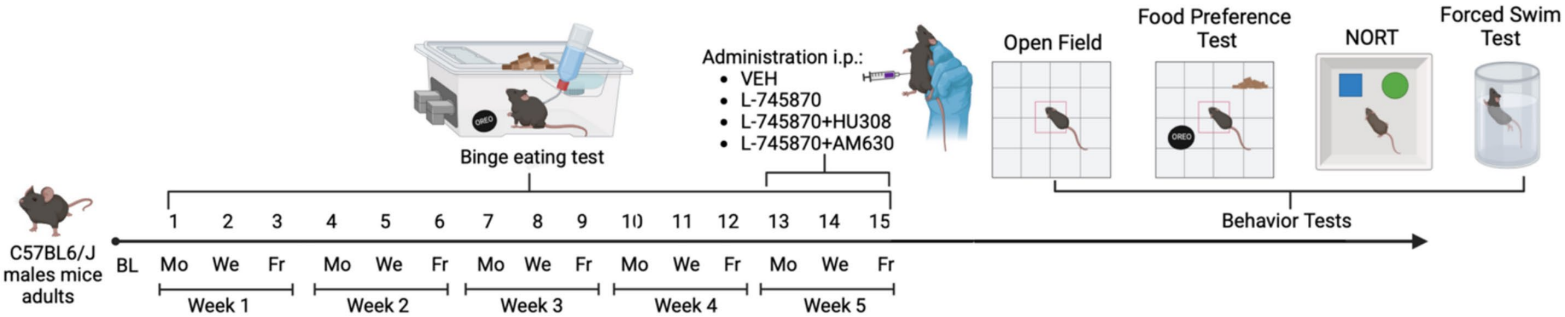
Experimental timeline. Mice received 15 binge test sessions with intermittent access to PF for 1 h on access days (Monday, Wednesday, and Friday) for 5 weeks (1st to 15th). On binge test sessions 13th to 15th, treatment was administrated: VEH, L-7458-70, L-7458-70 + HU308, and L-7458-70 + AM630. Animals had ad libitum access to SD and water. All experiments took place in the animal’s home cage. After the 15th binge test session, all animals were evaluated in behavioral tasks: Open Field, Novel Object Recognition Test (NORT), Food Preference Test, and Forced Swim Test. BL, baseline; Mo, Monday; We: Wednesday; Fr: Friday. Figure made with Biorender^®^ software.

Animals were evaluated in the behavioral tasks at the end of 15 BET sessions to assess the effects of the treatments administered (see Figure 1) in the following order: (1) open field test, (2) food preference test, (3) NORT, and (4) FST. All behavioral assessments were analyzed using ANY-maze^®^ Software (Stoelting Europe Co.^®^).

### 2.9 Statistical analysis

Data were prepared in Excel and are reported as the mean ± standard error of the mean (SEM). The results were analyzed using Graph Pad Prism version 9.3.1 (350) (GraphPad Software LLC, 2021). Figures were generated using GraphPad Prism^®^.

The PF binge-like intake is represented as the mean proportion of PF kilocalorie (kcal) intake per BET session, while body weight is represented as the mean weight per week in grams. We used a two-way ANOVA (group × weeks) with Sidak’s multiple comparisons test (∝ < 0.05) to examine the main effects of the treatment group (group) and time, as well as their interaction effect, on the following parameters:

- Body weight changes from BL week to Week 5.
- Binge-like intake: (a) BET sessions 1 to 12; (b) Comparison of BET sessions 1 vs. 12; (c) comparison of binge test sessions 13th to 15th (binge test sessions under treatment).

Furthermore, one-way ANOVA analysis with Sidak’s multiple comparisons test (∝ < 0.05) was used to assess differences between groups on body weight in Weeks 4 and 5.

Regarding behavioral tasks, results were analyzed as follows:

- OFT and FST: One-way ANOVA with Sidak’s multiple comparisons test (∝ < 0.05)
- FPT and NORT: Two-way mixed effects ANOVA (group × time) with Sidak’s multiple comparisons test (∝ < 0.05) to compare the main effects of treatment group and preference (exploration time).

## 3 Results

### 3.1 Palatable food intake increases across binge test sessions

**Figure 2:**
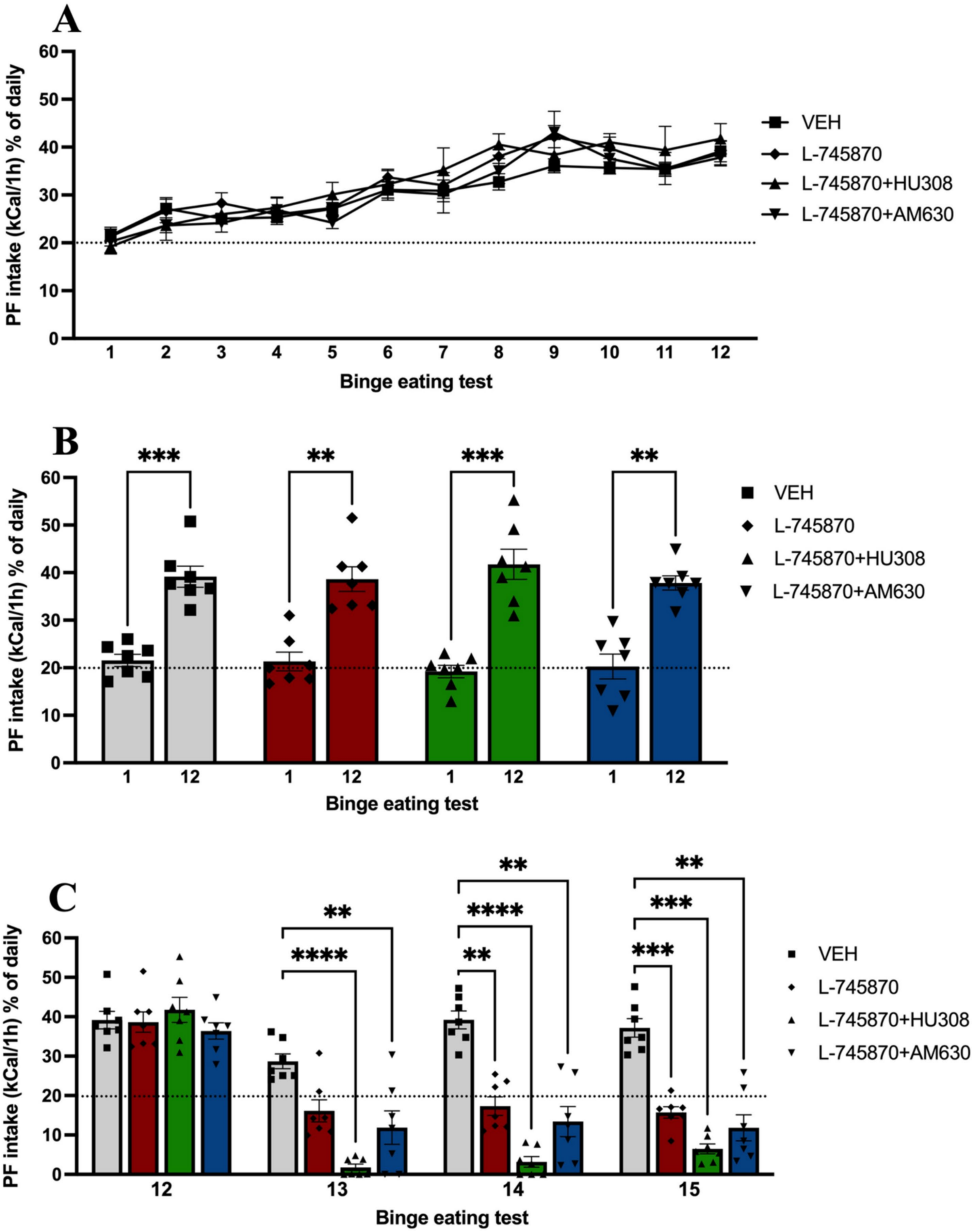
Proportion of PF caloric intake in binge test sessions. Data express the mean ± SEM (*n* = 7/group). **(A)** Point-plot of the PF intake in binge test sessions 1st to 12th. **(B)** The bar graph shows the bar plot of PF intake in binge test sessions 1st vs. 12th. **(C)** The bar graph shows the bar plot of PF intake in binge test sessions 12 to 15th and compares groups from binge test sessions 13th to 15th (binge test sessions under treatment). ****p < 0.0001, ***p < 0.001, **p < 0.01.

First, to determine the behavioral binge-like intake of PF was evaluated in adult mice for 12 baseline binge test sessions. Two-way ANOVA results showed no significant effect of group [*F*_(2.745, 16.47)_ = 1.196; *p* = 0.34] and interaction [*F*_(4.893, 29.36)_ = 0.8406; *p* = 0.53] in PF intake in binge tests 1 through 12; however, there was a significant effect of time [*F*_(4.576, 27.46)_ = 35.93; *p* < 0.0001], explaining 50.48% of variance, indicating that PF intake increased across time in binge tests, as shown in Figure 2A. Additionally, two-way ANOVA analysis with repeated measures was conducted to evaluate differences in PF intake between binge tests 1 and 12, which revealed significant differences in time between both sessions [*F*_(1, 6)_ = 198.5; *p* < 0.0001], explaining 70.63% of the total variance and indicating that all groups significantly increase PF binge-like intake when comparing sessions 1 and 12 (Figure 2B). Sidak’s multiple comparisons test indicates a significant increase in binge-like intake in all groups when comparing BET sessions 1 vs. 12 (all *p-*values < 0.01).

### 3.2 The effects of coadministration of DRD4 antagonist and CB2 agonist decreases binge-like intake

Previously, it has been shown that the administration of CB2R agonists reduces binge intake of PF in adolescent mice. Therefore, once binge-like intake was established and evaluated in baseline BET sessions, we evaluated the intraperitoneal coadministration of DRD4 antagonist and CB2 agonist or antagonist in the binge-like intake of PF for three additional BET sessions. Figure 2C shows the results of binge-like intake in BET sessions under treatment (13–15). Two-way ANOVA results indicate a significant effect of time [*F*_(1.705, 10.23)_ = 104.4; *p* < 0.0001], explaining 40.04% of total variance, treatment group [*F*_(1.589, 9.534)_ = 23.99; *p* = 0.0003], with 30.60% of variance explained, and interaction [*F*_(3.225, 19.35)_ = 12.67; *p* < 0.0001], explaining 12.80% of total variance. Sidak’s multiple comparisons test indicates significant differences in the following BET sessions:

- Thirteen, between the VEH and L-745870 + HU308 (*p* < 0.0001) and L-745870 + AM630 groups (*p* = 0.0059).
- Fourteen, between VEH and L-745870 group (*p* = 0.0013), L-745870 + HU308 (*p* < 0.0001), and L-745870 + AM630 groups (*p* = 0.0096), and
- Fifteen, between VEH and L-745870 (*p* = 0.0001), L-745870 + HU308 (*p* = 0.0001), and L-745870 + AM630 groups (*p* = 0.0069).

### 3.3 The effects of coadministration of DRD4 antagonist and CB2 agonist in anxiety, preference, memory, and depression tasks

**Figure 3:**
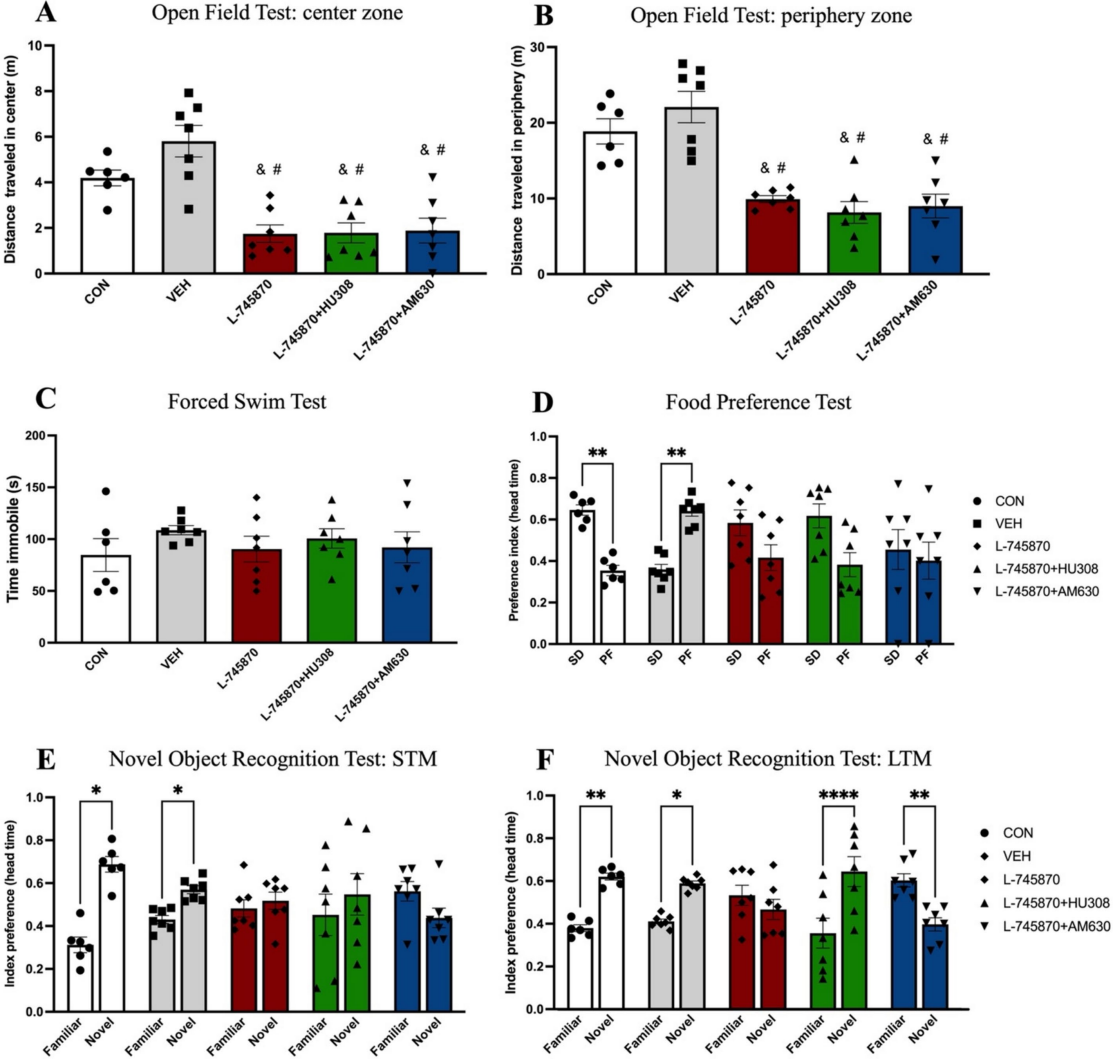
Effects of administration of treatments in behavioral tasks. Data express the mean ± SEM. **(A,B)** Open field test: the bar graph shows the bar plot of the distance traveled in the center **(A)** and in the periphery **(B)**. **(C)** Forced swim test: the bar graph shows the bar plot of immobility time. **(D)** Food preference test: preference index between SD and PF. **(E,F)** Novel object recognition test. The bar graph shows the bar plot of the recognition index between familiar and novel objects in short-term memory (STM) **(E)** and long-term memory (LTM) **(F)**. Data express the mean ± SEM. ****p < 0.0001; ***p < 0.001; **p < 0.01; *p < 0.01; & indicate a statistical difference (*p* < 0.01) between CON group; # indicate a statistical difference (*p* < 0.001) between VEH group.

To evaluate the effect of the coadministration on anxiety-like behavior, food preference, memory, and depressive-like behavior, we performed the following behavioral tasks: OFT, FPT, NORT, and FST. The results from behavioral tests are shown in Figure 3. One-way ANOVA results of OFT indicate a significant difference between groups in the distance traveled in the center [*F*_(4, 29)_ = 13.49; *p* < 0.0001; R2 = 0.6505]. Sidak’s multiple comparisons test indicates significant difference between CON and L-745870 (*p* = 0.0228), L-745870 + HU308 (*p* = 0.0262), and L-745870 + AM630 (*p* = 0.0369) groups; additionally, a significant difference was found between VEH and L-745870, L-745870 + HU308 and L-745870 + AM630 (all *p*-values <0.0001) groups (see Figure 3A). Furthermore, one-way ANOVA results of OFT indicate a significant difference between groups in distance traveled in the periphery [*F*_(4, 29)_ = 17.54; *p* < 0.0001; R2 = 0.7076]. Sidak’s multiple comparisons test indicates significant difference between CON and L-745870 (*p* = 0.0037), L-745870 + HU308 (*p* = 0.0004), and L-745870 + AM630 (*p* = 0.0012) groups, as well as a significant difference between VEH and L-745870, L-745870 + HU308 and L-745870 + AM630 groups (all *p*-values <0.0001) (see Figure 2B).

Regarding depressive-like behavior, as shown in Figure 3C, one-way ANOVA test results of FST show no significant differences among groups in time of immobility [*F*_(4, 29)_ = 0.6120; *p* = 0.657; R2 = 0.078].

Figure 3D shows the results of the food preference test. Two-way mixed effects ANOVA revealed a significant difference in preference index (row factor) between SD and PF [*F*_(1, 58)_ = 6.014; *p* = 0.017] and with group (column factor) × preference index (row factor) interaction [*F*_(2.217, 32.14)_ = 7.200; *p* = 0.002], while there was no significant effect of group (column factor) [*F*_(1, 14)_ = 0.5793; *p* = 0.46]. Sidak’s multiple comparisons test indicates that the CON group significantly prefers SD over PF (*p* = 0.009), while the VEH group significantly prefers PF over SD (*p* = 0.0064).

Finally, regarding memory evaluation, two-way mixed effects ANOVA results of NORT-STM indicate a significant effect in recognition index (row factor) between familiar and novel objects [*F*_(1, 6)_ = 6.795; *p* = 0.04] and interaction between group (column factor) and recognition index (row factor) [*F*_(1.780, 9.792)_ = 5.196; *p* = 0.03]; Sidak’s multiple comparisons test indicates that the CON (*p* = 0.017) and VEH (*p* = 0.047) groups significantly discriminate the novel object over the familiar (Figure 3E). Furthermore, two-way mixed effects ANOVA results of NORT-LTM indicate a significant effect in recognition between familiar and novel objects [*F*_(1, 6)_ = 10.60; *p* = 0.0019] and interaction [*F*_(4, 58)_ = 12.88; *p* < 0.0001]; Sidak’s multiple comparisons test indicates that the CON (*p* = 0.0018), VEH (*p* = 0.018), and L-745870 + HU308 (*p* < 0.0001) groups significantly discriminate the novel object over the familiar, while the L-745870 + AM630 (*p* = 0.0045) group significantly discriminate the familiar object over the novel (see Figure 3F).

### 3.4 Binge-like intake of palatable food modifies body weight in adult mice

**Figure 4:**
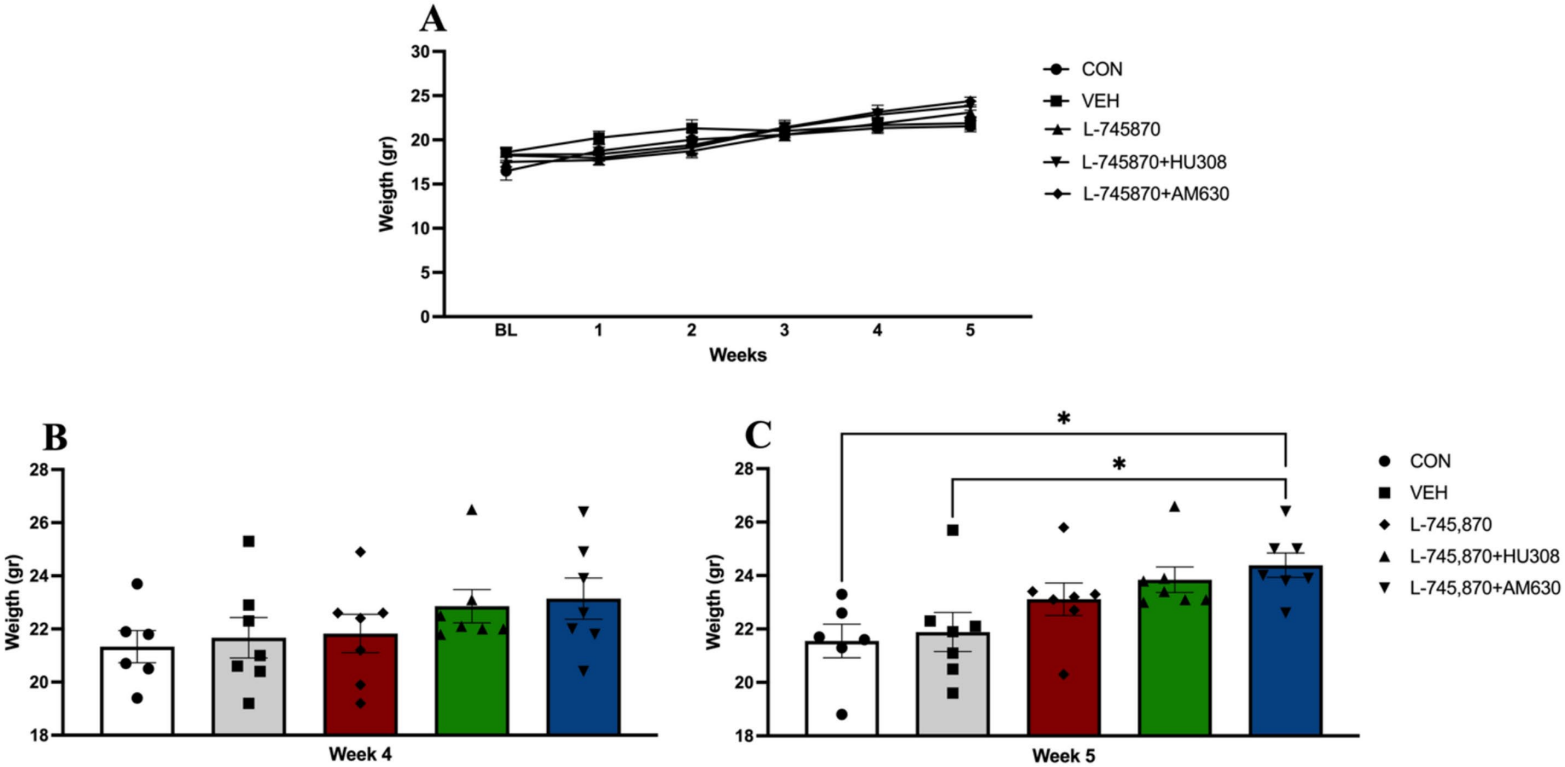
Body weight. Data express the mean ± SEM. **(A)** Body weight per week. **(B)** Body weight analysis between groups on Week 4 (before starting treatment). **(C)** Body weight analysis between groups on Week 5 (BET sessions under treatment); *p < 0.01.

To evaluate changes across time in body weight induced by binge-like intake of PF in adult mice from baseline week to Week 5, a two-way ANOVA analysis was conducted. The results indicate a significant effect of time [*F*_(2.48, 71.93)_ = 235.00; *p* < 0.0001], explaining 47.47% of total variance, and group x time interaction [*F*_(20,145)_ = 8.227; *p* < 0.0001], explaining 6.646% of total variance, while there was no significant effect of treatment group [*F*_(4,29)_ = 0.5327; *p* = 0.7127], with 2.705% of variance explained (see Figure 4A). Sidak’s multiple comparisons test indicates a significant difference between CON and L-745870 + AM630 groups in body weight in Week 5 (week of BET sessions under treatment) (*p* = 0.0473). Figures 4B,C show the results of body weight analysis between groups in Weeks 4 and 5 (BET under treatment). One-way ANOVA results of body weight in Week 4 indicate no significant differences between groups before starting treatment. Furthermore, one-way analysis results of body weight on Week 5 (BET sessions under treatment) revealed a significant difference between groups [*F*_(4,29)_ = 4.237; *p* = 0.0080]. Sidak’s multiple comparisons test indicates a significant difference between CON and L-745870 + AM630 groups (*p* = 0.0238) and between VEH and L-745870 + AM630 groups (*p* = 0.0473). This result on body weight may be explained by a compensatory effect of L-745870 increasing SD intake (Supplementary Figure 1) while decreasing the binge-like intake of PF by itself and in addition to CB2R agonism and antagonism.

## 4 Discussion

The present study aimed to evaluate the interaction between the endocannabinoid and dopaminergic system on binge-like intake of PF in mice. To evaluate this, mice were exposed to 12 binge-test sessions to induce binge-like eating and then were evaluated in three additional sessions under treatment with either a DRD4 antagonism alone or in combination with a CB2R agonist or antagonist. Our findings show that the present restricted intermittent access model reliably induces binge-like intake of PF in a non-caloric restriction-dependent manner, which is consistent with studies that suggest that PF consumption increases the risk of development of BED (Sinclair et al., 2015; Chávez-Hernández et al., 2024). Our results show that over 12 sessions of BET, there is a significant increase in binge-like intake episodes, with PF overconsumption escalating from 20 to 40% of total caloric intake, showing the driving of this maladaptive eating behavior.

The DA activity has been implicated in regulating eating behaviors (Gervasini et al., 2018). Furthermore, DRD4 has been identified to be involved in the modulation of the reward process of PF consumption and may contribute to the dysregulation of food intake in patients with eating disorders (Botticelli et al., 2020). Our results demonstrate that the administration of the DRD4 selective antagonists decreased binge-like intake of PF compared to control, which is consistent with other studies that show that the administration of DRD4 antagonists significantly reduced binge episodes (López-Alonso et al., 2023; Cruz-Trujillo et al., 2024), suggesting that this receptor plays a crucial role in facilitating the compulsive nature of binge eating. On the other hand, Yan et al. (2012) show that there was no effect in the administration of DRD4 antagonist in food-seeking behavior in rats in an operant paradigm; this inconsistency with our results may be explained regarding the task used and evaluation conditions. It is important to mention that the downregulation of DRD4 has been identified as a risk factor that may underly binge-purge syndromes in patients (Steiger et al., 2016). Additionally, a relationship between the DRD4 gene polymorphism and environmental influences has been proposed, suggesting that this interaction could play a key role in promoting overeating and weight gain (Botticelli et al., 2020). Together, our findings suggest that DRD4 signaling participates in the regulation of the consumption of PF, as well as the regulation of binge-like intake. Nevertheless, the systematic administration of DRD4 selective antagonists also impaired the memory process, a result that is consistent with previous studies (Miyauchi et al., 2017). In this regard, given our findings on the effect of DRD4 antagonism on memory and binge-like intake reduction, it is important that future studies focus on evaluating and differentiating these effects in relation to food palatability. Further research on the potential role of memory mechanisms, such as memory extinction, could provide valuable insights into how these processes may influence binge-like eating behavior.

One of the key findings of the present study indicates that the combination of DRD4 blockade with CB2 receptor activation induced the greatest reduction in binge eating compared to other treatments. This suggests a functional interaction between the dopaminergic and endocannabinoid systems in the modulation of binge-like eating behavior, potentially through CB2 receptor-mediated modulation of reward pathways to reduce this behavior. Although, initially, CB2R was considered a peripherally expressed receptor, such as in the spleen, leukocytes, testis, and muscle (Onaivi et al., 2012), CB2R activity has also been related to anti-inflammatory properties in both central and peripheral nervous system (García-Gutiérrez et al., 2025). In this regard, CB2R activation in microglial cells is associated with increased expression of anti-inflammatory factors such as IL-10 (Ma et al., 2015). In relation to this, our results show that systematic administration of a CB2R agonist may induce an anti-inflammatory effect. Even though this was not measured in the present study, it is important to determine it in future studies. Moreover, CB2R activity has been shown to reduce weight gain, relieve glucose tolerance, enhance insulin sensitivity, and attenuate inflammation by suppressing macrophage polarization in a mouse model of obesity (Wu et al., 2020). Furthermore, both receptors co-localize, such as neurons in the NAc (Zhang et al., 2014; Cruz-Trujillo et al., 2024). In this regard, studies suggest that CB2R activity participates in addictive behaviors such as cocaine self-administration behavior and sugar consumption by modulation of mesolimbic dopaminergic neurons (Zhang et al., 2017; Bourdy et al., 2021). Furthermore, CB2R activation has been suggested to regulate the reduction of VTA neurons’ excitability, the reduction of intracellular cAMP levels, and the enhancement of the M-type K + channel function (Ma et al., 2019). In this regard, studies have shown that DRD4 antagonists significantly reduced binge episodes (López-Alonso et al., 2023; Cruz-Trujillo et al., 2024) and that CB2R agonist reduces addictive behaviors, such as cocaine self-administration behavior and sugar consumption by modulation of mesolimbic dopaminergic neurons (Zhang et al., 2017; Bourdy et al., 2021; García-Blanco et al., 2023). Additionally, our group recently demonstrated that the administration of CB2R agonists reduces binge-like intake of PF (Rodríguez-Serrano and Chávez-Hernández, 2025). Furthermore, it has been suggested that CB2R are mainly expressed in the postsynapsis (Chen et al., 2017) and that their activity ultimately results in the suppression of neuronal activity. On the other hand, DRD4 has been identified in presynaptic neurons in the NAc, modulating excitatory neurotransmission (Svingos et al., 2000); also, it has been suggested that the stimulation of DRD4 modulates glutamatergic transmission in the NAc, promoting PF consumption (Cruz-Trujillo et al., 2024). Our results demonstrate that the combination of DRD4 antagonism and CB2R agonism effectively reduces binge-like intake of PF, presenting a novel potential therapeutic approach for BED. Furthermore, our results suggest an interaction effect between the blockade of DRD4 and the activation of CB2R.

On the other hand, our results show no significant reduction of binge-like intake by the coadministration of DRD4 antagonists with CB2R antagonists, suggesting that the activity of CB2R is key to reducing this behavior. In this regard, it has been reported that the lack of CB2R may constitute a protective factor, while overexpression may be a vulnerability for the development of food addiction (García-Blanco et al., 2023). Therefore, our results suggest that there is an interaction of DRD4 with CB2R in the regulation of binge eating behaviors. Additionally, results from the food preference test show that coadministration of DRD4 antagonist with either CB2R agonist or antagonist reduces exploration to PF compared to SD (non-significant), suggesting a decreased preference for PF that could be further explored behaviorally in future studies.

The DRD4 has been involved in the pathogenesis of neuropsychiatric diseases (Botticelli et al., 2020; Chestnykh et al., 2023). Therefore, we evaluated the effect of coadministration of DRD4 antagonist with CB2R agonist or antagonist in anxiety-like and depression-like behaviors. Our results show that the coadministration of DRD4 antagonist and CB2R agonist and antagonist reduced the distance traveled in the central and periphery of OFT compared to the CON and VEH groups. It is important to note that studies indicate that the administration of DRD4 antagonists does not produce locomotor alterations (Erlij et al., 2012). In this regard, our findings suggest that the treatments administered reduced anxiety-like behavior, evidenced by a reduction in the distance traveled in both areas of OFT, but this should be further explored in future studies. Furthermore, FST results show no difference between treatments, CON and VEH group, in immobility time, which suggests that the treatments administered did not induce depression-like behaviors. In this regard, it has been shown that the blockade of DRD4 (Basso et al., 2005) and activation of CB2R (Andersen, 2024) do not affect this type of behavior.

In summary, our results show that DRD4 may regulate eating behaviors, given that the administration of DRD4 antagonist decreases binge-like PF. Moreover, the combined effect of DRD4 receptor blockade with CB2 receptor activation produced an even more pronounced reduction in binge eating. These findings suggest an interaction between the dopaminergic and endocannabinoid systems, in which the activation of CB2 receptors further modulates the reward pathways and attenuates binge-like behavior. This mechanism could be further explored in future studies as a potential treatment for BED.

## Data Availability

The raw data supporting the conclusions of this article will be made available by the authors, without undue reservation.

## Glossary

PF: palatable food
BED: binge eating disorder
ECS: endocannabinoid system
AEA: anandamide
2-AG: 2-arachidonoylglycerol
CB1R: cannabinoid receptor 1
CB2R: cannabinoid receptor 2
ERK: extracellular signal-regulated kinases
AC: adenylate cyclase
VTA: ventral tegmental area
NAc: nucleus accumbens
DA: dopamine
DRD4: dopamine receptor D4
SD: standard diet
BET: binge eating test
Kcal: kilocalories
BL: baseline
OFT: open field test
NORT: novel object recognition test
STM: short-term memory
LTM: long-term memory
FST: forced swim test
FPT: food preference test
