# Supplementary material for: Coadministration antagonist dopamine receptor D4 with CB2 receptor agonist decreases binge-like intake of palatable food in mice
Source: Front Behav Neurosci. 2025 Apr 29;19:1572374. doi: 10.3389/fnbeh.2025.1572374 (PMC12069467; doi:10.3389/fnbeh.2025.1572374)
Supplement: Supplementary file 1 [file Table_1.docx]

Supplementary Material

## Supplementary Figures


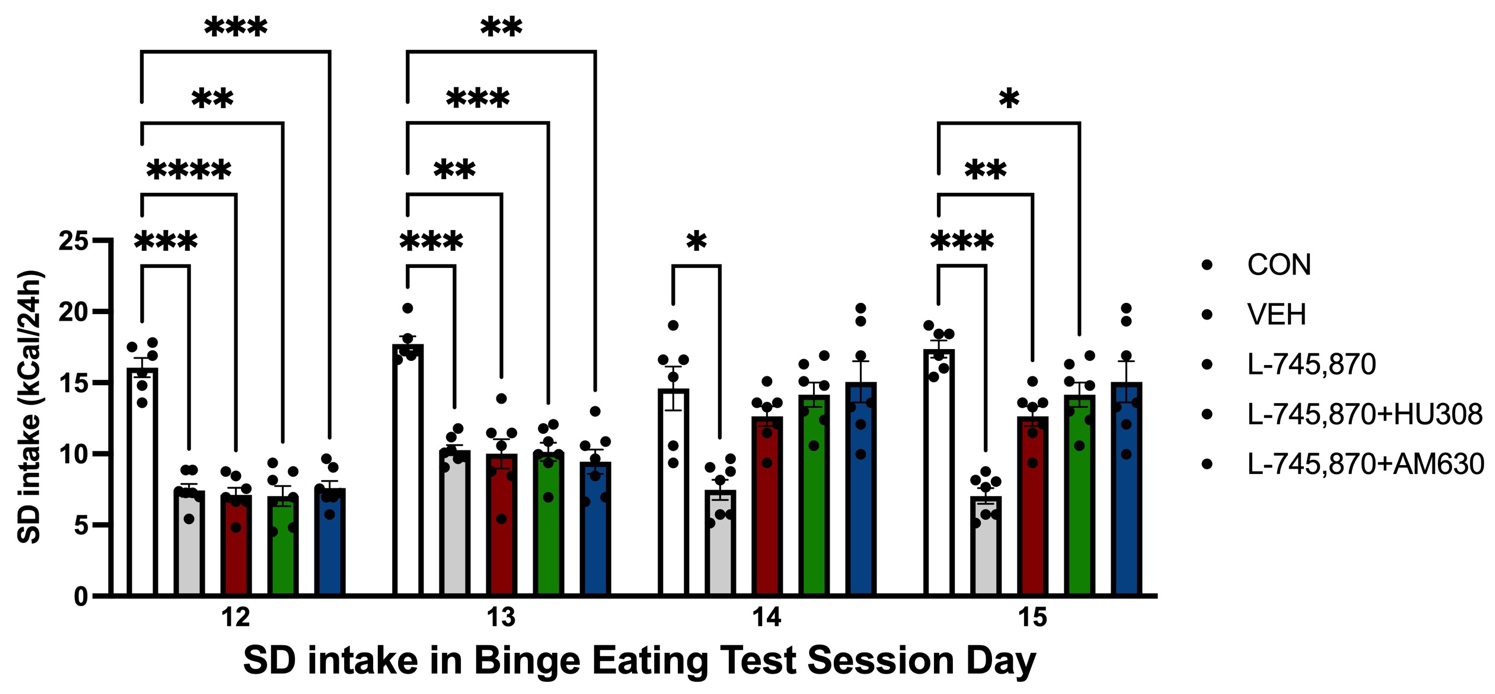


**Supplementary Figure**. Standard diet (SD) 24h kilocaloric (kcal) intake. Data express the mean ± SEM. Bar graph shows bar plot of SD intake in days when binge tests sessions 12 to 15 were conducted. Two-way ANOVA analysis (group × BET) with Sidak’s multiple comparisons test. *****p*<0.0001; ****p*<0.001; ***p*<0.01; ***p*<0.05.
